# Increased Alpha Band Functional Connectivity Following the Quadrato Motor Training: A Longitudinal Study

**DOI:** 10.3389/fnhum.2017.00282

**Published:** 2017-06-12

**Authors:** Stefano Lasaponara, Federica Mauro, Filippo Carducci, Patrizio Paoletti, Mario Tombini, Carlo C. Quattrocchi, Carlo A. Mallio, Yuri Errante, Laura Scarciolla, Tal D. Ben-Soussan

**Affiliations:** ^1^Cognitive Neurophysiology Laboratory, Research Institute for Neuroscience, Education and Didactics, Patrizio Paoletti Foundation for Development and CommunicationAssisi, Italy; ^2^Department of Neuropsychology, IRCCS Fondazione Santa LuciaRome, Italy; ^3^Department of Psychology, Sapienza Università di RomaRome, Italy; ^4^Department of Physiology and Pharmacology, Sapienza Università di RomaRome, Italy; ^5^Unit of Neurology, Neurophysiology, Neurobiology, Department of Medicine, Università Campus Bio-Medico di RomaRome, Italy

**Keywords:** functional connectivity, sensorimotor training, longitudinal studies, quadrato motor training, interhemispheric communication, eLORETA

## Abstract

Quadrato Motor Training (QMT) is a new training paradigm, which was found to increase cognitive flexibility, creativity and spatial cognition. In addition, QMT was reported to enhance inter- and intra-hemispheric alpha coherence as well as Fractional Anisotropy (FA) in a number of white matter pathways including corpus callosum. Taken together, these results seem to suggest that electrophysiological and structural changes induced by QMT may be due to an enhanced interplay and communication of the different brain areas within and between the right and the left hemisphere. In order to test this hypothesis using the exact low-resolution brain electromagnetic tomography (eLORETA), we estimated the current neural density and lagged linear connectivity (LLC) of the alpha band in the resting state electroencephalography (rsEEG) recorded with open (OE) and closed eyes (CE) at three different time points, following 6 and 12 weeks of daily QMT. Significant changes were observed for the functional connectivity. In particular, we found that limbic and fronto-temporal alpha connectivity in the OE condition increased after 6 weeks, while it enhanced at the CE condition in occipital network following 12-weeks of daily training. These findings seem to show that the QMT may have dissociable long-term effects on the functional connectivity depending on the different ways of recording rsEEG. OE recording pointed out a faster onset of Linear Lag Connectivity modulations that tend to decay as quickly, while CE recording showed sensible effect only after the complete 3-months training.

## Introduction

Quadrato Motor Training (QMT) is a new training paradigm, which was found to increase cognitive flexibility and attention in contrast to simple motor training (SMT) and mental training (Ben-Soussan et al., 2013, 2015, 2017). As a motor task, QMT involves a series of cognitive and mental operation, which required dividing attention between the motor response for producing the correct sequences of movement and the current location of the body within the Quadrato Space. As advantages, QMT is a relatively short train and can be easily practiced in limited spaces, and in comparison to other whole-body training paradigms, it can be quantified in terms of accuracy and reaction time.

Due to its unique nature, in the recent years QMT has been deeply investigated in order to highlight eventual behavioral and physiological changes induced by this whole-body-training. Previous studies have shown that QMT improves reaction times, cognitive flexibility and spatial cognition, in contrast to SMT or verbal training following a session or a month of daily QMT training (Ben-Soussan et al., 2013, 2015). QMT was further found to improve emotional well-being (Ben-Soussan et al., 2015) and in addition, QMT increased emotional regulation following a month of daily training, in contrast to a SMT (Ben-Soussan, 2014) and to have greater effects as compared to breathing meditation (Paoletti et al., in press).

At the electrophysiological level, these results were matched with enhanced inter- and intra-hemispheric alpha (8–12 Hz) coherence (Ben-Soussan et al., 2013). Moreover, using resting state Magnetic Resonance Imaging (rsMRI) and Diffusion Tensor Imaging (DTI) it was pointed out that 12-weeks training of daily QMT lead to increased Fractional Anisotropy (FA) in a series of white matters pathways including corpus callosum, anterior thalamic radiations, corticospinal tracts, cerebellar peduncles and superior longitudinal fascicule (Ben-Soussan et al., 2015).

Taken together, these results seem to suggest that electrophysiological and structural QMT-induced changes may be due to an enhanced interplay and communication of the different brain areas within, but especially between the right and the left hemisphere. In particular, alpha oscillation and alpha band functional connectivity could be the target of sensorimotor processes (Neuper et al., 2006; Sabate et al., 2011, 2012; Lopes da Silva, 2013), also as they are closely involved in motor execution, motor imagery and visuomotor accuracy (Rilk et al., 2011; Sabate et al., 2011). In addition, the consistent report related to increased clustering of alpha networks has led to the model suggesting that alpha oscillation could carry elaborate information about the neurophysiological ongoing processes assuming also the role of local neuronal processing (Athanasiou et al., 2012, 2014, 2016).

A non-invasive method that allows measuring the way different brain areas communicate with each other, is the resting state (rs)-functional connectivity.

The use of functional connectivity is usually expressed with linear dependence (coherence) and nonlinear dependence (phase synchronization) covariation between fluctuations in activity recorded from distinct neural networks. These measures are normally defined as the sum of lagged dependence and instantaneous dependence. Coherence and phase synchronization are interpreted as “connectivity” between locations. However, under normal circumstances, any measure of dependence is highly contaminated with an instantaneous, non-physiological contribution due to volume conduction and low spatial resolution. Using exact low-resolution brain electromagnetic tomography (eLORETA) software allows to considerably removing this confounding factor (Pascual-Marqui, 2007). For this reason, in the recent years this software has been used to evaluate non-invasive functional connectivity measures, as respect with various EEG bands in different task and different fields of investigations both in psychological and neuroscientific studies such as the ones concerning: major depression (Olbrich et al., 2014), cognitive decline (Vecchio et al., 2014), food-addiction (Imperatori et al., 2015) and mindfulness meditation (Lehmann et al., 2012).

In the present study, using the same technique, we aim to replicate and furtherly explore previous finding concerning QMT, evaluating its longitudinal effects on the functional connectivity measures of the alpha-band recorded during a resting state electroencephalography (rsEEG) with both open (OE) and closed eyes (CE) conditions, in a large sample of healthy participants undergoing 3-months of daily QMT.

A further confirmation of this hypothesis can be found in a recent work by Paoletti and Ben-Soussan (2016) in which inter- and intra-hemispheric functional connectivity was measured in Mild Cognitive Impairment (MCI) patients undergoing 1 month of daily QMT or control Walking Motor Training (WMT). Through the use of eLORETA, the authors successfully reported that, compared with the WMT, the QMT induced an enhancement of the functional connectivity from right occipital to right parieto-temporal source activity at alpha 1 and alpha 2 frequency bands. Noticeably, decreased functional connectivity of these rsEEG rhythms characterizes Alzheimer’s Disease (AD) patients (Jelic et al., 2000; Knott et al., 2000; Almkvist et al., 2001; Adler et al., 2003; Babiloni et al., 2016).

## Materials and Methods

### Participants

We investigated 50 healthy volunteers (27 women and 23 men, 35 ± 5 years and 36 ± 6 years). Inclusion criteria were: (1) age between 25 and 45 years; (2) right-handedness; (3) no medical history that might affect the EEG measures (history of traumatic injury, current or past drug or medication addiction/abuse, antidepressant medications use, recent depressive episodes); (4) absence of motor impairment, emotional and behavior disorders (EBD), general cognitive disorders or developmental coordination disorder (DCD, aka Dyspraxia) and; (5) negative history of previous practice of the QMT.

Exclusion criteria were: (1) history of traumatic injury, previous neurosurgery, stroke, inflammatory/infective diseases of the brain; (2) co-morbidity of congenital metabolic diseases or malformations; (3) diagnosis of one histologically proven primary cancer (<1 years); (4) Vitamin B12 deficiency, positive serology for secondary dementia (RPR/ VDRL, HIV, anti-Borrelia), abnormal thyroid function considered significant during clinical examination; (5) clinical evidence of depression (assessed with the Geriatric Depression Scale ≥ 14) or other psychiatric conditions, epilepsy, drugs or alcohol addiction (according to DSM IV-TR); (6) severe impairment of cognition (Mini Mental State Examination ≤ 24); (7) subjects already included in a motor activation program; (8) diagnosis of malnutrition (based on body mass index); (9) chronic inflammatory disease (e.g., Rheumatoid Arthritis) and other diseases in the acute phase; (10) hearing or visual impairment or motor deficits incompatible with the workout (according to the physician opinion); (11) treatment with hormone replacement therapy; and (12) current or recent history of smoking (i.e., not smoking during the last year). Participants were instructed of the option to interrupt the QMT and dropout from the study at any time for any reason including change in the clinical status deemed incompatible with the continuation of the study, refusal to continue with the study protocol and personal needs.

From the starting number of subjects, we excluded three subjects because they were left-handed, four subjects because of white matter lesion load, seven subjects because of MRI/EEG incomplete protocol and two subjects because of lack in complying motor exercise. Thus, 34 healthy right handed subjects were selected for the EEG and behavioral analyses (20 females).

### Ethics Statement

The ethical committee of the Università Campus Bio-Medico di Roma, Rome, Italy, approved the study (09/14 PAR ComEt CBM). The experimental protocol was designed as a controlled experimental phase I study. Data were collected in compliance with GCP (Good Clinical Practice) and following the ALCOA (Attributable, Legible, Contemporaneous, Original and Accurate) algorithm. The TREND checklist was accomplished (S1 TREND Checklist).

### Procedure

In the first visit, the participants were introduced to the facilities and all procedures were explained, adequate understanding was tested and written informed consent was obtained from the participants in accordance with the Declaration of Helsinki. Then several cognitive tests were assessed. Subsequently, participants underwent an EEG 20-channel EEG recording and finally performed the Quadrato Motor Exercise on the Quadrex platform.

**Figure 1:**
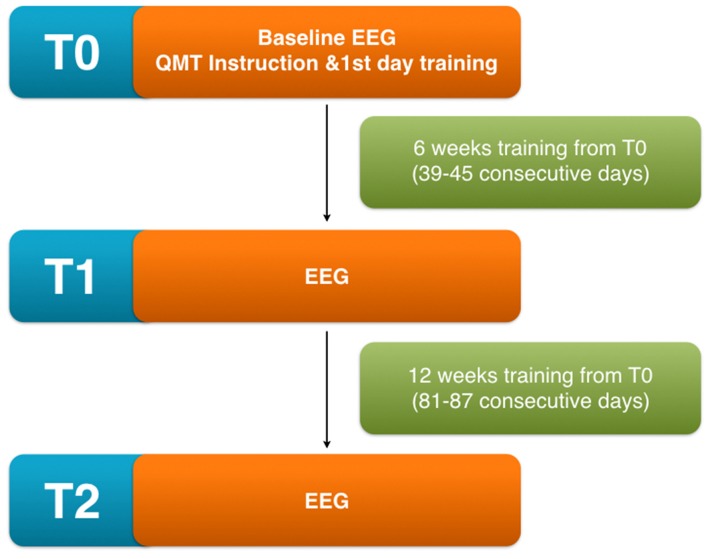
Scheme of the experimental protocol showing the different time points involved in the analysis T0 (before initiating quadrato motor training (QMT) training), T1 (after 6 weeks from the beginning), T2 (after 12 weeks from the beginning).

At each time point, every volunteer was also requested to show the accuracy and the completeness of the diary to the researchers as a pre-requisite to undergo next time point measurements. Participants were informed of the option to interrupt the QMT and drop-out from the study at any time for any reason including: change in the clinical status deemed incompatible with the continuation of the study, refusal to continue with the study protocol and personal needs (see Figure 1).

### The Quadrato Motor Training (QMT)

The QMT requires standing at one corner of 0.5 m × 0.5 m square and making movements in response to verbal instructions given by an audio tape recording (for additional details, see Ben-Soussan et al., 2013). In the QMT there are three optional directions of movement. The instructions direct participants to keep the eyes focused straight ahead and hands loose at the side of the body. They were also instructed to immediately continue with the next instruction and not to stop in the case of mistakes. At each corner, there are three possible directions to move in, thus the training consists of 12 possible movements. In this longitudinal experimental protocol, the daily training consisted of a sequence of 69 commands lasting 7 min. We used a movement sequence paced at a rate of an average of 0.5 Hz (similar to a slow walking rate) and instructed the participants to begin all movements with the leg closest to the center of the square.

### EEG Recording and Analysis

EEG was recorded in wake rest state (5 min with eyes-closed and 5 min with eyes open) in late morning hours from 19 electrodes positioned according to the International 10–20 System (i.e., Fp1, Fpz, Fp2, F7, F3, Fz, F4, F8, T7, C3, Cz, C4, T8, P7, P3, Pz, P4, P8, O1, O2) with common average reference (time constant of 0.3 s, 0.3–70 Hz filtering bandpass, MICROMED Brain Quick System). To monitor eye movements, the vertical electro-oculogram was simultaneously recorded. All data were digitized in continuous recording mode (256 Hz sampling rate). Impedances of all electrodes were kept below 5 kΩ. In order to keep constant the level of vigilance, an operator controlled on-line the subject and the EEG traces, alerting the subject any time there were signs of behavioral and/or EEG drowsiness.

The EEG data pre-processing was performed via the EEGLAB (Delorme and Makeig, 2004) toolbox for MATLAB. Data were re-referenced to the average reference, low-pass filtered (cut off 45 Hz) and high-pass filtered (cut off 0.5 Hz) and then divided in 1 s epochs. All trials were visually inspected for artifacts and eye movements and potentially rejected. Moreover, they were subjected to an algorithm that automatically rejected epochs when the signal exceeded by abnormal spectra (±50 dB in the 0–2 Hz band for eye movements and ±25 to 100 Hz in the 20–40 Hz for muscular activity), by probability (±3 standard deviations from the mean distribution of occurrence of each trial) and by extreme values (±70 μV).

### Low-Resolution Brain Electromagnetic Tomography (eLORETA)

We used eLORETA to localize the generators of the scalp EEG power spectra and for the evaluation of the functional connectivity. The eLORETA solution space is restricted to the cortical gray matter in the digitized MNI atlas with a total of 6239 voxels at 5 mm spatial resolution (Pascual-Marqui et al., 2002). The fundamental assumption of LORETA directly relies on the neurophysiological observation of coherent firing of neighboring cortical neurons. Cross-validation studies using simultaneous functional magnetic resonance imaging have provided evidence for a high validity of LORETA localization findings (Mulert et al., 2004, 2005). The eLORETA assumes that the smoothest of all activity distributions is the most plausible, and it is based on images of standardized current density. The unique property of eLORETA in contrast to the former LORETA version is that under ideal conditions, eLORETA has zero localization error (Pascual-Marqui, 2002). One downfall of the method is that eLORETA does not filter noisy measurements, so its usage is constrained to artifact-free data.

**Figure 2:**
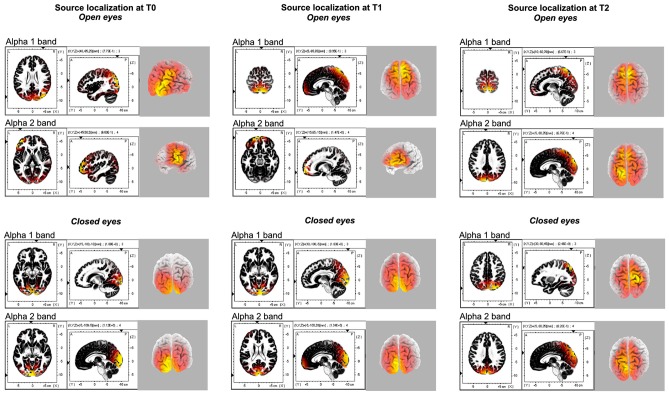
Source localization of the different alpha 1 and alpha 2 band generators for each time points (T0, T1, T2) and experimental conditions (Open eyes OE, Closed eyes, CE).

We calculated tomographic eLORETA images corresponding to the estimated neuronal generators of brain activity within a given frequency range using default values provided by LORETA free software: δ (1.5–6.0 Hz), θ (6.5–8.0 Hz), α1 (8.5–10.0 Hz), α2 (10.5–12.0 Hz), β1 (12.5–18.0 Hz), β2 (18.5–21.0 Hz), β3 (21.5–30.0 Hz) and Ω (>30 Hz). A spatial over-smoothing with signal-to-noise ratio 10 was chosen for the eLORETA transformation matrix. This procedure resulted in one 3D LORETA image for each subject and experimental condition for a given frequency range. Successively 3D LORETA images were averaged across subjects resulting in one average 3D LORETA maps for each experimental condition. These are reported in Figure 2.

Finally, following the procedure adopted in previous studies (Babiloni et al., 2016) the eLORETA solutions at the voxel level were also averaged in cortical macro-regions of interest (ROIs). We considered six macro-regions, namely the frontal, central, parietal, occipital, temporal and limbic ROIs. Table 1 reports the Brodmann areas (BAs) included in these ROIs.

**Table 1:** Brodmann areas included in the region of interest (ROI) used for the exact low-resolution brain electromagnetic tomography (eLORETA) analysis of linear lagged connectivity.

Brodmann areas included in each of eLORETA ROI
Frontal	8, 9, 10, 11, 44, 45, 46, 47
Central	1, 2, 3, 4, 6
Parietal	5, 7, 30, 39, 40, 43
Temporal	20, 21, 22, 37, 38, 41, 42
Occipital	17, 18, 19
Limbic	31, 32, 33, 34, 35, 36

### Estimation of Functional Connectivity of rsEEG Cortical Sources

Functional connectivity between two cortical regions has previously been defined as the non-linear and linear dependence, as lagged non-linear and linear coherence of intra-cortical EEG-source estimates (Pascual-Marqui, 2007). When computed in the cortical source space, the inherent low spatial resolution of the EEG tomography enters high phase synchronization and zero-lag coherence (Pascual-Marqui, 2007). Activity at any cortical area is observed instantaneously (zero-lag) by all scalp electrodes. Instantaneous coherence between two cortical sources might also be found when a third source has an impact on two other brain sources (“common feeding”) even whether the two paired sources do not influence each other or as there is activity at reference electrode in coherence analysis.

For this reason, only the lagged coherence contribution of these measures should be considered for more save neurophysiological considerations. In this line, Pascual-Marqui (2007) proposed the solution to remove the zero-lag instantaneous interactions and to compute coherence using the residual, corrected time series. Furthermore, the proposed solution included measures of dependence among multivariate EEG time series (Pascual-Marqui, 2007). This procedure of functional connectivity called lagged linear connectivity (LLC) was implemented to make it available a freeware in the LORETA package (Pascual-Marqui et al., 2011). We used this original algorithm for the analysis of the rsEEG data in the present study. For each subject and frequency band (i.e., delta, theta, alpha 1, alpha 2, beta 1, beta 2 and gamma), the LLC was computed for six ROIs (i.e., frontal, central, parietal, occipital, temporal and limbic). For the inter-hemispherical analysis, the LLC estimates were calculated between all voxels of the mentioned ROIs of each hemisphere with the corresponding ones of the other hemisphere (homolog) and also with the remaining five ROIs. The LLC solutions for all voxels of a given pair of ROIs were averaged. For the intra-hemispherical analysis, the LLC estimates were computed for all voxels of a particular ROI with all voxels of another ROI of the same hemisphere. The LLC solutions for all voxels of a given pair of ROIs were averaged. This operation was repeated for the left and the right hemisphere.

## Statistical Analysis

Since previous studies investigating electrophysiological correlates of the QMT pointed out a selective modulation of alpha-band in power spectrum, coherence and connectivity (Ben-Soussan et al., 2013, 2015), we had focused our *post hoc* analysis and consideration only on significant effects concerning the two sub-alpha components alpha 1 and alpha 2, although all the different bands were analyzed. In order to evaluate the longitudinal effects of the QMT over rsEEG, we computed statistical analysis on both the current density and the LLC values according to the following procedure.

### Current Density

The current density values indicating source localization for each of the different eight EEG-bands considered in the study were analyzed using two different approaches, investigating both the whole brain level and at the ROI level. Whole brain analyses were performed through the statistical tools included in the LORETA software package. The methodology used is Statistical non-parametric Mapping (SnPM). It is based on estimating, via randomization, the empirical probability distribution for the max-statistic (e.g., the maximum of a *t* or an *F* statistic), under the null hypothesis. This methodology corrects for multiple testing (i.e., for the collection of tests performed for all voxels, and for all discrete frequencies). Due to the non-parametric nature of the method, its validity need not rely on any assumption of Gaussianity.

In this case, we ran a series of paired *T*-test with frequency scale normalization and smoothing 1, comparing the current density value in each voxel between all the experimental conditions. More specific, for both the OE and the CE, the sequent contrasts were computed: T1 > T0; T2 > T0; T2 > T1. Successively, for each time point we also contrasted OE with Closed Eyes (CE). The significance threshold was based on a permutation test with 5000 permutations. The t-values were plotted onto a MRI template with a scale bar indicating statistical power and color scale.

At the ROI level, the values of current density for each macro-region were exported and inserted in a repeated measures ANOVA with factors: EYES (Open, Close) × FREQUENCY (δ, θ, α1, α2, β1, β2, β3 and Ω) × TIME POINT (baseline, following 6- and 12 weeks of training; T0, T1 and T2, respectively) × ROI (Frontal, Central, Parietal, Temporal, Occipital and Limbic) × HEMISPHERE (Left, Right).

### Linear Lag Connectivity (LLC)

LLC was investigated separately for inter and intra-hemispheric connectivity both for the OE and CE conditions. In the case of inter-hemispheric connectivity, first the LLC values were analyzed considering the connectivity between homologs areas through a repeated measure ANOVA with factors: ROI (Frontal, Central, Parietal, Temporal, Occipital and Limbic) × TIME POINT (T0, T1 and T2) × FREQUENCY (δ, θ, α1, α2, β1, β2, β3 and Ω). Second, we also investigated the possible connection between each ROI among the six considered in the first hemisphere with each of the five ROIs remaining (excluding the homolog) in the second hemisphere. This was done through a series of six (one for each ROI) repeated measures ANOVA with factors: TIME POINT (T0, T1 and T2) × FREQUENCY (δ, θ, α1, α2, β1, β2, β3 and Ω) × ROI PAIR.

Intra-hemispheric connectivity was investigated separately for the left and the right hemisphere. We used the same procedure described above, with the only exception that each ROI was compared with the other five ROIs within the same hemisphere, through a series of 12 (one for each ROI, in the left and in the right hemisphere) repeated measures ANOVA with factors: TIME POINT (T0, T1 and T2) × FREQUENCY (δ, θ, α1, α2, β1, β2, β3 and Ω) × ROI PAIR.

## Results

### Source Localization of the α Band

#### Eyes Open Condition

Source localization of α band (8–12.5 Hz) activity was performed for the two sub-component α1 (8–10 Hz) and α2 (10–12 Hz) bands for all of the three different time points (T0-T1-T2).

During baseline α1 band selectively peaked in the right posterior areas (i.e., superior occipital gyrus B.A. 19; MNI coord: 40, −85 and 25), while α2 oscillation was more pronounced in the left middle frontal gyrus (B.A. 10; MNI coord: −45, 50 and 5).

A similar source localization pattern was found for the T1 condition. Also in this case, α1 band selectively peaked in the right posterior areas (i.e., precuneus B.A. 7; MNI coord: 5, −65 and 65), and α2 was more pronounced frontally, in the left superior frontal gyrus (B.A. 11; MNI coord: −15, 65 and −15).

Finally, the source localization in the T2 condition (Figure 2) pointed out that both the peaks of α1 and α2 activities were located in the posterior cortex, although in different areas, namely in the in the right Postcentral gyrus (B.A. 7; MNI coord: 10, −60 and 70) and in the left Cuneus (B.A. 19; MNI coord: −5, −90 and 35), respectively. By the way, a minor peak for the α1 was found in the right middle frontal gyrus (B.A. 10; MNI coord: 45, 55 and −6).

#### Eyes Closed Condition

As computed for the OE, also for the CE the source localization of α band (8–12.5 Hz) activity was performed for the two sub-component α1 (8–10 Hz) and α2 (10–12 Hz) bands for all of the three different time points (T0-T1-T2). In T0, α1 band peaked medially in the right posterior areas (i.e., lingual gyrus B.A. 18; MNI coord: 15, −100 and −10), while α2 oscillation was more pronounced in the left cuneus (B.A. 17; MNI coord: −5, −100 and −5). A similar source localization pattern was found for the T1 time point. In this case, both α1 and α1 bands peaked in the cuneus, respectively in the right (B.A. 17; MNI coord: 10, −100 and −5), and in the left (B.A. 17; MNI coord: −5, −100 and 20) hemisphere.

Finally, the source localization in the T2 condition evidentiated peaks of α1 frequency in the right superior parietal lobule (B.A. 7; MNI coord: 30, −80 and 45). Conversely, α2 activities were located in the posterior cortex, specifically in the left cuneus (B.A. 19; MNI coord: −5, −90 and 35).

### Current Density

#### Longitudinal Effect of QMT

##### Whole brain results

In order to investigate longitudinal effect of QMT on the current density, we compared separately all the different time points, both in the OE and in the CE experimental conditions.

Whole brain results concerning the two sub-component of the α band showed no modulation by the time points on the source localization/current density of these two frequencies. These negative results were observed both in the OE and in the CE.

##### ROI results

In line with what was observed at the whole brain level, ROI results were distinguished by the absence of any main effect or interaction involving the factor TIME POINTS (All *F* < 2).

### Linear Lag Connectivity

#### Inter-Hemispheric Connectivity

**Figure 3:**
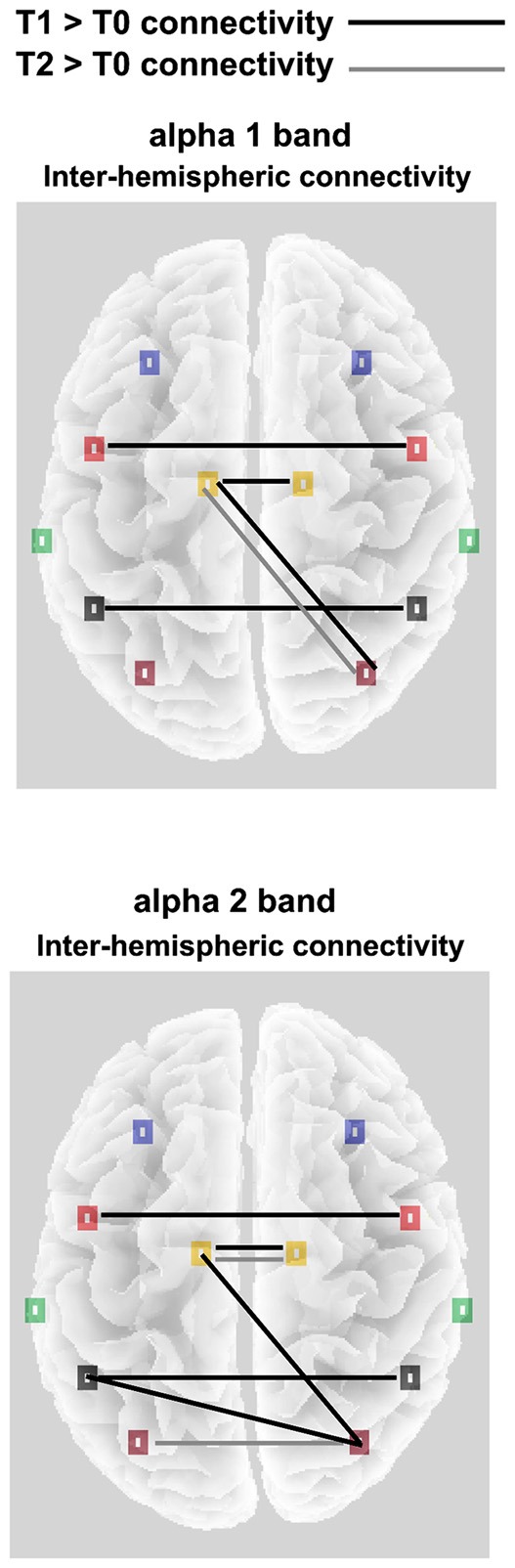
Significant alpha 1 and alpha 2 inter-hemispheric lagged linear connectivity (LLC) values for the OE experimental condition; Black lines indicates T1 > T0 significant connectivity; Gray lines indicates T2 > T0 significant connectivity.

As reported in Figure 3, in the OE condition a significant ROI × TIME POINTS × FREQUENCY interaction (*F*_(70,2310)_ = 1.3, *p* < 0.05), pointed out an increased homolog area connectivity between T1 and T0, both for the α1 and the α2 frequencies, in the Central, Parietal and Limbic ROIs. Conversely, an enhanced connectivity between T2 and other time points was only observed in the Occipital (compared to T1) and Limbic ROIs (compared to T2).

Concerning the inter-hemispheric connectivity between non-homologs areas, we only found a significant TIME POINTS × FREQUENCIES × ROI PAIR interaction in the comparison between occipital vs. all the other ROIs. This interaction (*F*_(70,2310)_ = 1.4, *p* < 0.01; see Figure 3) showed an enhanced connectivity in T1 as compared to T0, for the α1 band between occipital and limbic ROIs. In the same comparison, α2 frequency showed increased LLC values in the connection between occipito-parietal and occipito-limbic areas. Finally, T2 only showed increased connectivity as compared to T0 for the α1 band, again in the occipito-limbic comparison. Finally, in the CE experimental condition, no significant main effect or interaction was found in the ANOVA comparing LLC values, both in the homologs and non-homologs tests, indicating a lack of connectivity effect in each direction in this condition.

#### Intra-Hemispheric Connectivity: Left Hemisphere

**Figure 4:**
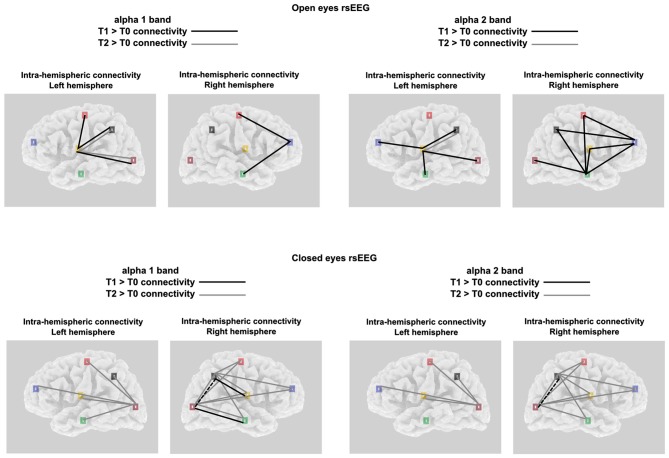
Significant alpha 1 and alpha 2 intra-hemispheric LLC values for both the experimental conditions and the brain hemispheres; Black lines indicates T1 > T0 significant connectivity; Gray lines indicates T2 > T0 significant connectivity.

**Figure 5:**
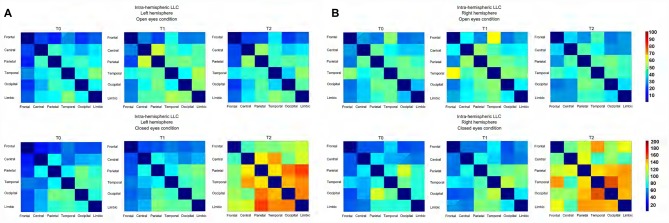
**(A)** Connectivity matrix showing the intensity, as a function of color, of the intra-Left-hemispheric LLC values relative to alpha oscillation (including both alpha 1 and alpha 2) for both the experimental condition. **(B)** Connectivity matrix showing the intensity, as a function of color, of the intra-Right-hemispheric LLC values relative to alpha oscillation (including both alpha 1 and alpha 2) for both the experimental condition.

Considering the intra-hemispheric connectivity in the left hemisphere, we found in the OE condition a significant TIME POINTS × FREQUENCIES × ROI PAIR interaction, only in the ANOVA comparing limbic vs. all other ROIs (*F*_(56,1848)_ = 1.4, *p* < 0.05; see Figures 4, 5A). This showed that between T0 and T1, α1 frequency-LLC increased in the limbico-central, limbico-parietal and limbico-occipital while α1 frequency-LLC was greater in all of the limbico comparison except limbico-central pair. If we consider T2, we found no difference in LLC of α1 or α2 bands as compared to T1, and minor differences as compared to T0 in limbico-parietal and limbico-occipital ROI pairs.

A completely different pattern was observed in the CE condition. Here, a significant TIME POINTS × FREQUENCIES × ROI PAIR interaction was selectively found in the ANOVA comparing occipital vs. all other ROIs (*F*_(56,1848)_ = 1.5, *p* < 0.001; see Figures 4, 5A). In this experimental condition LLC values were particularly similar in T0 and T1, and increased in T2. In particular, the ANOVA indicated an increased connectivity of α1 band in all of the ROIs pairs considered. The same happened for the α2 band, with the only exception of the occipito-temporal comparison.

#### Intra-Hemispheric Connectivity: Right Hemisphere

In the right hemisphere, for the OE condition the interaction of interest TIME POINTS × FREQUENCIES × ROI PAIR, was found to be significant in the ANOVA comparing frontal vs. all other ROIs (*F*_(56,1848)_ = 1.6, *p* < 0.001; see Figures 4, 5B) and also in the one comparing temporal vs. all other ROIs (*F*_(56,1848)_ = 1.3, *p* < 0.05; see Figures 4, 5B). Taken together, these interactions showed that, while no difference was observed between connectivity in T2 and T0, an increased LLC was observed in T1. In particular, *Post hoc* pointed out an higher connectivity of α1 band among fronto-central and fronto-temporal ROI pairs and an higher connectivity of α2 band in all of the temporo- ROI pairs and in fronto-central and fronto-parietal comparison.

Similar to what was observed in the left hemisphere, in the right hemisphere, in the CE condition we found a general greater enhancement in connectivity in T2 time point, as compared to both T0 and T1. By the way, here we also reported some significant differences in T1 vs. T0 comparison. These effects were revealed by the significant interaction TIME POINTS × FREQUENCIES × ROI PAIR, which was present both, in the ANOVA comparing parietal vs. all other ROIs (*F*_(56,1848)_ = 1.3, *p* < 0.05; see Figures 4, 5B) and occipital vs. all other ROIs (*F*_(56,1848)_ = 1.4, *p* < 0.05; see Figures 4, 5B). T2 time point *post hoc* indicated that LLC values related to α1 and α2 bands were higher in all of the parieto- and temporo- ROIs pairs. Conversely, T1 vs. T0 *post hoc* indicated increased α1 connectivity in Parieto-limbic and Occipito-temporal pairs, but also a decreased connectivity of both α1 and α2 (red asterisk) in Parietal vs. Occipital comparison (for similar results see Paoletti and Ben-Soussan, 2016).

## Discussion

The aim of the present study was to investigate the longitudinal dynamic electrophysiological changes induced by the 3-months QMT to rsEEG, comparing OE and CE conditions.

Using conventional EEG approaches it was already demonstrated that QMT increases alpha coherence, as well as ideational flexibility (Ben-Soussan et al., 2013, 2015). Here we focused on the electrophysiological changes over time, taking into account eventual differences in source generators of the alpha band and its functional connectivity measured with the eLORETA software.

In agreement with these previous results, the source localization pointed out that the main cortical areas involved in the alpha oscillation induced by QMT on the rsEEG with OE, were the left anterior and right posterior areas. Interestingly, the use of the eLORETA source localization algorithm allowed to highlight a dissociation between the two sub-component of alpha 1 and alpha 2, being accountable respectively of the posterior and the anterior oscillations. Conversely, the rsEEG with CE showed no differences between these alpha component and consequently between the source localization of the alpha band activity located in the posterior occipito-parietal cortex.

By the way, we should report that these spatial differences in source localization did not correspond with differences in the generators of the power spectrum (i.e., standardized current densities values). Using two different approaches, we analyzed the longitudinal differences induced by the sequent session of QMT (T0, T1, T2) in both OE and CE experimental condition. A first approach consisted in the whole brain analysis through the statistical tools included in the LORETA software package. In the OE-rsEEG, these analyses pointed out no significant results in the comparison between the three different time points, both for the alpha 1 and alpha 2 spectral values generators. Also for the CE-rsEEG we reported no significant results for what concern alpha 1 and alpha 2 band.

As a second approach, we analyzed the average current densities values extracted in each of six macro-region covering the entire brain, defined as ROIs. Also in this case the ANOVA pointed out not significant effects of the factor time points, showing no differences in the strength of the oscillation of the two alpha sub-component between the various time points.

Taken together, these results seem to suggest that there are no differences induced by longitudinal QMT sessions on the alpha subcomponent generators measured as current density values in the whole brain level or at the ROI level. Conversely, QMT as previously demonstrated (Ben-Soussan et al., 2013) may have an effect on the dynamic electrophysiological changes as indexed by alpha coherence or functional rs-connectivity.

This latter hypothesis seems to be confirmed by the Linear Lagged Connectivity analysis. Here we took advantage of the LLC values extracted from each of the six experimental ROI and we measured eventual changes in inter- and intra- hemispheric connectivity as a function of training period.

The first important results concerning inter-hemispheric connectivity regard the lack of effect in the CE experimental condition. On the other hand, in the OE for both the alpha 1 and 2, we found an increased connectivity between homologs Central, Parietal and Limbic ROIs, and more important, an increased connectivity for the alpha 1 in occipito-limbic areas and for alpha 2 in the occipito-parietal pathways.

The occipito-limbic circuit links the occipital cortex and amygdala, and is usually activated in response to emotional processing (Morris et al., 1998; Catani et al., 2003; Pavuluri and Sweeney, 2008). Interestingly, this network is dysfunctional in affective disorders and impulsivity (Pavuluri et al., 2012). In contrast, QMT increases reflectivity (as opposed to impulsivity) and reduces impulsivity and automatic responses as requires the ability to wait for up-coming command in an attentive way (Ben-Soussan et al., 2015). Thus, the current results are in line with previous behavioral results demonstrating that QMT significantly improves emotional regulation and well-being (Ben-Soussan et al., 2015). For example, QMT was previously found to increased emotional regulation following a month of daily training, in contrast to a SMT in university students (Ben-Soussan, 2014). In addition, the effect of QMT on emotional regulation was studied utilizing the Affect Balance Scale (ABS), comparing two intense 1-week training programs: (1) Breathing Meditation retreat with QMT training (*n* = 42); and (2) Breathing Meditation retreat without the QMT (*n* = 42). While both groups reported improved affect and self-efficacy following the training, the QMT group reported significantly higher ABS scores following the training (Paoletti et al., in press).

Concerning the intra-hemispheric connectivity, we reported noticeable differences across the two hemispheres and between the two experimental conditions. In the OE condition, we found that after the first session of QMT (i.e., T1), limbic ROIs of the left hemisphere increased its alpha connectivity with all of the other ROIs of the same hemisphere while in the right hemisphere this led to an increased connectivity of the alpha band between temporal and frontal areas with all of the other cortical ROIs involved in the analysis. Conversely, the second session of QMT did not improved furtherly the connectivity leading to weaker effects, resulting in enhanced connectivity of alpha 1 (and in minor part) of the alpha 2, only in the left hemisphere and in particular in the limbico-parietal and limbico-occipital ROIs pairs. A similar pattern seems to suggest that: (1) QMT with OE seem to have inverted-U curve effects in alpha connectivity; (2) These effects seem to be related more to the right than to the left hemisphere.

The current results are thus in line with previous research on skill learning, which tends to show evidence for an inverted-U-shaped learning curve due to reallocation of cognitive resources in other training paradigms, such as motor skill learning (Doyon et al., 2002), and language expertise (Sakai, 2005), as well as attentional expertise in meditation (Slagter et al., 2007). In fact, Slagter et al. (2007) found that activation in a network of brain regions typically involved in sustained attention showed an inverted u-shaped curve in which expert meditators (EMs) with an average of 19,000 h of practice had more activation than novices, but EMs with an average of 44,000 h had less activation. However, whether these activation differences are due to skill learning or strategy and task performance differences cannot be definitely resolved in the current study.

This lateralization factor instead, is consistent with EEG studies demonstrating changes in right hemispheric bias in alpha power changes (Quandt et al., 2012) as well as with functional neuroimaging research showing a right-sided bias in processing movement, object-related actions and gestures (Chaminade et al., 2005; Weiss et al., 2006).

A very different scenario was present in the CE experimental condition. Here, we generally observed the highest increment in alpha connectivity after 12-weeks of daily QMT (i.e., T2), with small differences observed after the first session. More specific, in the left hemisphere we reported an increased connectivity between occipital areas and the other five ROIs only in T2. Similarly, in the right hemisphere the second QMT session produced the greatest effect in the enhancement of alpha-connectivity in both parieto- and temporo- ROI pair comparisons. In this case, some differences were present also in the T1 vs. T0 comparison; indeed, we found an increment in alpha1 connectivity after the first session of QMT in occipito-temporal connections. These results are in agreement with a recent finding reporting inter- and intra- connectivity enhancement after QMT (Ben-Soussan et al., 2013), but especially with another study reporting the same occipito-temporal enhanced connectivity in MCI patients undergoing 1 month-training of QMT as compared to MCI patients without the same training (Paoletti and Ben-Soussan, 2016). In the light of the results of the present study, future directions could involve longer training for MCI patients in order to show greatest effect, that in our case were observed after 2 months of QMT.

Taken together, these results seem demonstrating different effects on the functional connectivity induced by QMT, depending on the state during which rs-EEG was recorded. Major effects were observed with CE after complete 3-months training, while with OE the greatest effects on the connectivity were reached after half of the total training.

One could hypothesize that increased alpha connectivity in the CE condition, and the inverted-U curve in the OE condition may be related to the different states to which each condition is related. Previous investigations already showed differences in topography and power of alpha oscillation between EC and EO (Barry et al., 2007; Lasaponara et al., 2016); while other studies suggested that EC and EO states may reflect the difference of information communication (Tan et al., 2013; Miraglia et al., 2016). Similarly, in the context of the present study, one may argue that CE alpha reflects internal oriented attention and desynchronization could be indicative of the engagement in cognitive processes and external attention (Benedek et al., 2014). The OE condition may be related to more engagement with the external world. Thus, the difference between open and CE condition following training, may reflect as state of going into internal oriented attention.

Concerning the specific involvement of alpha oscillations in sensorimotor processes, previous studies showed changes in alpha power according to participants’ expertise in various disciplines (Hatfield et al., 1984; Collins et al., 1990; Salazar et al., 1990; Crews and Landers, 1993; Radlo et al., 2002), also during resting state conditions (Liu et al., 2003; Vernon, 2005). Looking at the phase-locked activity, it has been suggested that alpha activity could promote the cortical processing of information increasing phase-locked and decreasing non-phase-locked activity (Sabate et al., 2011). Interestingly, previous studies showed how the QMT could lead to an increase of alpha coherence, suggesting that this result could be a direct effect of the complexity of the training, which require a great combination of mind-body information (Ben-Soussan et al., 2013). More specifically, when looking at alpha functional connectivity, it is noteworthy that previous studies reported an increased clustering, in the context of a model where alpha rhythm elaborates and transmits information on the neurophysiological processes, assuming the role of local neuronal processing (Athanasiou et al., 2012, 2014, 2016).

To conclude, QMT differentially induces functional connectivity modifications in different networks, mostly related to emotional regulation and spatial attention depending on the condition. Interestingly, the changes related to emotional regulation were obtained in the OE condition, while the changes in the occipital networks were obtained in the CE condition, suggesting an interaction between internal and external attention. Future studies should continue this line of research, also comparing different training paradigms, such as internalizing and externalizing disorders, such as depression and impulsivity.

## Author Contributions

TDB-S, FC, MT and CCQ designed the research; MT, CCQ, CAM, LS and YE performed the research; SL and FM analyzed the data; SL, FM and TDB-S wrote the article; FC, MT, CCQ and PP contributed to the writing process.

## Conflict of Interest Statement

The authors declare that the research was conducted in the absence of any commercial or financial relationships that could be construed as a potential conflict of interest.
